# Notes and News

**Published:** 1891-04-18

**Authors:** 


### Notes and News.

Collected and Communicated.

The influenza is subsiding in Chicago, though there were 951 deaths last week. It was a grim sort of 'cuteness which led our Yankee cousins to take advantage of the epidemic to have a "corner" in coffins. When the demand became great the undertakers did not fail to raise their prices, and it became a very serious matter to die, for there was the possibility that you could not be buried. Not that coffins are a necessity by any means; there is every reason why we should prefer to go straight to mother-earth when the spirit of life has left us, but public opinion is at present in favour of a box instead of a martial or other cloak, and so one reads with wonder of a "corner in coffins."

The annual report of the Ambulance Committee of the Asylums Board for the last year states that all the removals of patients to the hospitals have been effected with entire freedom from accident. By the land service the total number of fever patients removed was 8,235, a considerably larger number than in any previous year. This high figure appears to have been due, not to any exceptional prevalence of disease, but to the assistance given to the local sanitary authorities by the immediate intimation of the existence of cases of disease which they now receive under the provisions of the "Infectious Diseases (Notification) Act, 1889." The river ambulance steamers conveyed 428 patients and other passengers. The cost of the land service was £7,076 19s. 4d., and of the river service, £3,824.

At the meeting of the Home Hospitals Association held on Tuesday, the 14th inst., at the Home Hospital, Fitzroy House, Fitzroy Square, the Chairman, Sir Richard Quain, Bart., F.R.S., traced the gradual building up of this, the first pay hospital which had been successful. Starting with one house and fourteen beds, it had soon increased to two houses and twenty-four beds, a nurses' home being afterwards added with a staff of twelve private nurses, shortly to be augmented by four more. During time years that the institution has been in existence 2,141 patients have been treated who have paid for the benefits they have received no less than £40,000. The institution is saddled with a mortgage and loan of some £6,000, which eats up about £230 a-year, and the Chairman remarked that if some benevolent individual could be found to pay this off, that £230 a-year would be available towards lessening the charges to those who could ill afford to pay them. Although there is a small balance on the wrong side on the year's working, the report of the Committee states that the financial condition of the Association is satisfactory, but the Committee regret that they do not see their way this year to pay off any of the debt. They, however, are glad to report that a substantial decrease has taken place in several items in the expenditure account. All present spoke in high terms of the efficiency of the establishment under the excellent management of Miss Judkins, the Lady Superintendent, and Mr. T. Almond Hind, the Hon. Secretary.

Dr. John Chapman is not idle in his exile in Paris, for he is still publishing books and publicly preaching the principles of Neuro-Dynamic medicine. To many the evils of sea-sickness, dyspnœa, &c., appear scarcely less bearable than the cure by an ice-bag to the spine—it makes one shudder only to think of it. Yet the results of this treatment are very remarkable, as Professor Peter testified lately in his course on Pathology at l'Ecole de Médecine.

An extraordinary case has just been recorded by a surgeon of a man who has lived for thirty-eight years with a pin embedded in his larynx. The pin was swallowed by accident when the man, now aged forty-five, was only seven years old. Sharp pain was felt at the time, but this soon passed away, the man keeping in good health for twenty-four years. He then began to be troubled by constant cough and huskiness of voice, which no remedy would relieve. After a violent fit of coughing one day, a dark, hard object was expelled, which proved on examination to be the long-lost and forgotten pin. All symptoms disappeared as soon as it had been expelled, and the man made a good recovery.

The quarterly statement of the Devonshire Hospital and Buxton Bath Charity gives a satisfactory account of the work done during the first three months of the year. 314 in-patients have been admitted, of whom 308 have been discharged as improved. The annual subscriptions received have been in considerable excess beyond those received during the corresponding quarter of the previous year, and the total ordinary receipts during the quarter have been more than those of the first three months, of 1890, by £106 6s. 7d. The authorities of this hospital have the satisfaction of knowing that its sanitary arrangements are in accordance with the most modern ascertained requirements, nothwithstanding that is the result of converting a building that had been used as a great range of stabling during seventy years into a hospital to receive 300 patients.

At the annual meeting of the supporters of St. Mary's Hospital, Manchester, the Chairman, Mr. Duncan Matheson, announced that an advantageous site for the proposed new buildings had at last been found. After patient enquiries and deliberations the Committee had secured a site in Oxford Street, which meets with the entire approval of the Medical Committee and the principal subscribers. The designing and building of the hospital have been entrusted to Mr. Alfred Waterhouse. The plans will be drawn out for the original scheme of 100 beds, but fearing to cripple the future resources of the hospital by a heavy debt the Committee do not feel justified in proceeding with the entire building, and a portion containing, perhaps, 60 beds will be erected, leaving a section, or wing, to be completed when funds permit.

The one hundred and ninth annual meeting of the Governors of the Nottingham General Hospital, was held under the presidency of Earl Manners, on the 8th inst. The report for 1890 states that special attention has been given to the sanitary arrangements of the hospital, and extensive repairs and improvements have been undertaken, and when they are completed the sanitation of the hospital will be as good as the structural arrangements of the old buildings will allow. The work has been heavier than usual during the year; the daily average of patients being 140, one of the highest on record. The financial statement shows a decrease in the number of the subscribers, and the receipts amount to rather less than last year's. After the adoption of the report, Mr. Stiebel proposed to publish in the local papers the names of all the residents paying as much as £100 a year for rent, who did not subscribe to the hospital; in reply to which suggestion Mr. Enfield asked whether Mr. Stiebel made the proposition with a view to popularising the institution, and said he wished to express his strongest feeling against anything likely to be so mischievous.

